# Human Uterine Decidual NK Cells in Women with a History of Early Pregnancy Enhance Angiogenesis and Trophoblast Invasion

**DOI:** 10.1155/2020/6247526

**Published:** 2020-02-18

**Authors:** Ningyi Jia, Jian Li

**Affiliations:** Beijing Obstetrics and Gynecology Hospital, Capital Medical University, Beijing, China

## Abstract

**Objective:**

The present study aimed to identify changes in decidual natural killer (dNK) cells and related cytokines in women who have undergone induced abortions (IAs). The effects of dNK cells on subsequent pregnancies remain unknown. Accordingly, we sought to investigate whether a history of early pregnancy can change dNK cells and facilitate their role in the regulation of angiogenesis and trophoblast invasion. *Materials and Methods*. dNK cells were obtained from primiparous women who had undergone IA(s) prior to this study and primiparous women who had never been pregnant before this IA (control). Real-time polymerase chain reaction (PCR) was used to measure the mRNA levels of IFN-*γ*, IP-10, VEGF, and PLGF in dNK cells. The levels of these cytokines were quantified using the enzyme-linked immunosorbent assay. HUVEC and HTR-8/SVneo cells were used to evaluate the angiogenesis, migration, and invasion activities influenced by dNK cells.

**Results:**

In dNK cells, the mRNA level of IFN-*γ*, IP-10, VEGF, and PLGF in dNK cells. The levels of these cytokines were quantified using the enzyme-linked immunosorbent assay. HUVEC and HTR-8/SVneo cells were used to evaluate the angiogenesis, migration, and invasion activities influenced by dNK cells.

**Conclusion:**

The findings of this study suggest that a history of early pregnancy has an impact on dNK cells. These trained dNK cells can regulate angiogenesis and trophoblast invasion and migration by promoting the production of certain cytokines.

## 1. Introduction

Placentation in the first trimester substantially affects reproductive success [1]. Research has shown that normal pregnancy and delivery will protect the subsequent pregnancies. First pregnancies are linked to lower birth weights and increased risk of pregnancy disorders such as preeclampsia (PE) [2]. It has been reported that repeated pregnancies can train the memory of decidual natural killer (dNK) cells leading to their enhanced function in subsequent pregnancies [3]. In a previous study, single-cell reconstruction of the early maternal-fetal interface was performed and it was verified that the initiation of dNK1 cells during the first pregnancy responds more effectively in subsequent pregnancies [4]. The dNK cells account for 70% of the decidual immune cells with the capacity of producing cytokines, but limited cytotoxicity. Previous reports suggested that dNK cells might be involved in decidualization, angiogenesis [5], regulation of trophoblast invasion [6], and spiral artery remodeling [7].

IA is a remedial method to deal with contraceptive failure. Although IAs are generally safe and effective, the side effects and influence on subsequent pregnancies should not be ignored. According to previous studies, repeated IAs may cause uterine adhesion, placenta previa, and pelvic inflammation, which impact subsequent pregnancies [8]. However, studies have shown a lower risk of PE associated with IAs in primiparous women [9–13]. A history of light endometrium injuries, such as biopsy and curettage may increase the success rate of implantation in assisted reproductive techniques [14]. The underlying mechanism of these findings has not been elucidated so far. However, it could possibly be attributed to the inflammatory response that contributes to decidualization and implantation. Furthermore, surgical abortions may be associated with endometrium injuries and subsequent spiral artery remodeling, which may be associated with the pathogenic pathway of PE. It was observed that the invasiveness of a trophoblastic cell can be increased by decidual injury [15]. Additionally, longer interpregnancy intervals can lead to a higher risk of PE [11, 16]. It seems that among women with a prior IA or shorter interpregnancy interval, maternal immunological recognition of trophoblast may be improved, which may contribute toward placentation and uteroplacental perfusion. Parker et al. [12] speculated that the inflammatory response to IA improves placentation and reduces the risk of PE. However, the pregnancy itself may change the uterine immunological environment, which may contribute to the maternal-fetal crosstalk in the subsequent pregnancy.

To date, there is no evidence showing a relationship between IA or early pregnancy and changes of maternal-fetal interface. Gamliel et al. [3] found that repeated pregnancies were associated with improved placentation and pregnancy-trained dNK cells might contribute to improved placentation. Therefore, this study aimed to identify changes in the function of dNK cells and the related cytokines in women who have undergone IA. We also sought to investigate whether the dNK cells are trained during the first trimester of gestation. Additionally, we intended to elucidate the role of dNK cells in the regulation of trophoblast invasion and angiogenesis.

## 2. Materials and Methods

### 2.1. Study Population

All tissue samples were collected with informed consent according to the requirements of the Research Ethics Committee of Beijing Obstetrics and Gynecology Hospital, Capital Medical University (Beijing, China). The inclusion criteria were as follows: (1) pregnancy with a gestational age of 6–9 weeks; (2) no prior births; (3) fetal heartbeat detectable through ultrasound. The exclusion criteria were as follows: (1) women with a history of missed abortion, medical abortion, or delivery; (2) severe inflammation (pelvic inflammatory disease or endometriosis); (3) severe liver or kidney disease; (4) immunological disease; (5) ultrasound confirmed that there was no fetal heartbeat. Decidual samples (*n* = 33) were obtained from healthy women whose pregnancies were terminated for nonmedical reasons at 6–9 weeks of gestational age. Seventeen of these individuals had never been pregnant before this IA (age, 25 ± 2.57 years; gestational age, 7.41 ± 0.78 weeks). The other 16 participants had no history of delivery but had undergone IA(s) prior to the study (age, 27.44 ± 5.45 years; gestational age, 7.44 ± 0.80 weeks; number of IAs, 1.25 ± 0.58). The tissue fragments derived from the decidua after surgically induced abortions were placed into a container with 25 mL of sterile Roswell Park Memorial Institute (RPMI, Gibco, Grand Island, NY, USA) 1640 medium.

### 2.2. Uterine Flush Sample Collection

After sterilization, the cervix was cleansed with 0.9% bacteriostatic sodium chloride to remove mucus from the cervical canal. The uterus was then flushed with 5 mL of sterile saline solution using an artificial insemination catheter. Ultrasound was used to supervise the whole process. Uterine flush samples (*n* = 10) were collected and then centrifuged to confirm cytokine expression in vivo.

### 2.3. Isolation of Decidual Cells

Decidual tissues derived from the first trimester surgical abortions were washed in sterile phosphate-buffered saline (PBS, Gibco, Grand Island, NY, USA). After the clearance of blood, the tissue was minced and digested with 0.1% collagenase and 100 IU/ml DNase I by shaking in a 37°C water bath for 30 min. Fetal bovine serum (FBS, Gibco, Grand Island, NY, USA) was added to stop the digestion followed by centrifugation. The resulting pellet was resuspended in complete RPMI containing 20 *μ*g/ml penicillin/streptomycin and then seeded onto Petri dishes followed by overnight incubation at 37°C in a humidified 95%O_2_ : 5% CO_2_ incubator to enrich decidual cells.

### 2.4. Isolation and Culture of dNK Cells

The decidual NK cells were filtered and resuspended in RMPI 1640. The leukocyte-enriched supernatants were centrifuged and cell pellets resuspended in complete RPMI, layered over Ficoll-Hypaque and centrifuged. Decidual cells were centrifuged (1800 rpm, room temperature) for 30 min in Ficoll gradients (MP Biomedical, Santa Ana, CA, USA) to collect the mononuclear cells. Ficoll layers were collected and resuspended in RPMI 1640. The supernatant was then filtered. Aliquots of 10^7^ cells were resuspended in 40 *μ*l MACS buffer followed by the incubation of cells with a CD56+ microbead cocktail at 4°C for 15 min. Microbeads (Miltenyi Biotec, Bergisch Gladbach, Germany) were used for the selection of dNK cells. The cells were centrifuged and resuspended in MACS buffer solution, and then loaded onto a column positioned in a magnetic separator (MACs; Miltenyi Biotec, Bergisch Gladbach, Germany). Cells were separated with MACS cell separation columns. Flow cytometry was used to examine the purity of dNK cells.

### 2.5. Real-Time Polymerase Chain Reaction (PCR)

Real-time PCR was used to measure the mRNA levels of IP-10, VEGF, PLGF, and IFN-*γ* in dNK cells. TRIzol reagent (Tiangen Biotech, Beijing, China) was used to extract the total RNA from 1 × 10^6^ dNK cells. The RNA samples were assessed for their purity and concentration by spectrophotometry. The cDNA was synthesized from 500 ng of total RNA using PrimeScript RT Master Mix (TaKaRa, Japan) in a total volume of 10 *μ*l at 37°C for 15 min, 85°C for 5 s, and stored at −20°C before use. Real-time PCR was performed based on the detected fluorescence with SYBR Premix Ex Taq (TaKaRa, Japan). The RNA levels were used in the mRNA levels of the standard. PCR was performed using specific primers synthesized by Invitrogen (Table 1). Relative mRNA expression levels were calculated using REL = 2^−ΔΔCt^, where ΔCt = Ct target gene-Ct 18SrRNA. All experiments were performed in triplicate.

### 2.6. Enzyme-Linked Immunosorbent Assay (ELISA)

The culture medium from the dNK cells was collected and stored at −80°C for cytokine measurements. The levels of cytokines (IFN-*γ*, IP-10, VEGF, and PLGF) in the medium were quantified by ELISA based on kit instructions (R&D Systems, Minneapolis, MN, USA). All measurements were performed in triplicates to minimize the influence of technical error and intra-assay variation.

### 2.7. Transwell Invasion and Migration Assays

After coculturing with dNK cells, HTR-8/SVneo and HUVEC cell lines were used to evaluate invasion and migration. Transwell assays were performed to assess the influence of dNK cells from each group on the invasion and migration potential of HTR-8/SVneo cells. Approximately 200 *μ*l of HTR-8/SVneo cells (at a concentration of 5 × 10^5^ cells/ml) suspension was plated in invasion chambers, which were immersed in 24-well cell culture plates containing the supernatant of dNK cells from each group. The plates were cultured at 37°C with 5% CO_2_. Noninvading cells were removed from the top of the Matrigel (BD Biosciences, San Jose, CA, USA) by cotton swabs after 24 h. The cells that had invaded the Matrigel were fixed using 4% paraformaldehyde and dyed with 0.1% crystal violet. The average number of invading cells in five random fields at 100x magnification was calculated for each insert.

We assessed the migratory behavior of HTR-8/SVneo cells and HUVECs incubated with dNK using Transwell assays. Cells were plated on the well (5 × 10^5^ cells/ml) and incubated with the supernatant of dNK cells from each group. The migrated cells were then counted using a light microscope (Olympus, IX51, Japan).

### 2.8. Tube Formation

After coculturing with dNK cells, HUVEC suspension was added to each well of a 96-well BD BioCoat angiogenesis plate. Approximately 20 *μ*l of HUVECs (5 × 10^5^ cells/ml) suspension was placed in 96-well plate with 20 *μ*l of supernatant from dNK cells and incubated at 37°C with 5% CO_2_. We evaluated the extent of tube formation microscopically after 6 h.

### 2.9. Statistical Analysis

The means and standard deviations or medians and interquartile ranges for variables were calculated. Since the data did not meet the assumptions for normality, nonparametric tests (Mann–Whitney *U* test) were used for comparisons. *p* value less than 0.05 was considered to be significant. SPSS software (SPSS for windows version 23.0; SPSS Inc., Chicago, IL,USA) and GraphPad Prism 8.0 (GraphPad Software, La Jolla, CA, USA) were used to perform statistical analyses.

## 3. Results

### 3.1. Differences in the Secretion Function and Level of mRNA in dNK Cells of the Control and IA Groups

**Figure 1:**
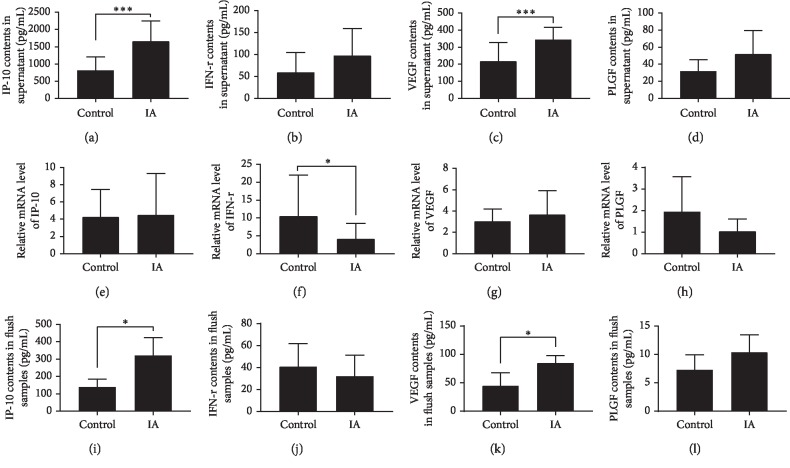
Cytokines secretion and mRNA levels in dNK cells of two groups. Cytokines secretion in uterine flushing samples.

The dNK cells can secrete cytokines to affect pregnancy. Through ELISA and Real-time PCR, we detected the secretion of four cytokines in the supernatant and their mRNA levels in dNK cells. The results demonstrated that more IP-10 and VEGF were secreted in the supernatants of dNK cells isolated from women with a history of IAs compared to those in the control group (*p* < 0.01). The mRNA level of IFN-*γ* in dNK cells was higher in the control group than that in the IA group (*p* < 0.05). However, the relative levels of PlGF, IP-10, and VEGF mRNA in dNK cells of both groups were not significantly different between the two groups (Figure 1).

### 3.2. Differences in Cytokine Expression Levels between Uterine Flush Samples from Control and IA Groups

Because cytokine expression differs between in vivo and in vitro environments, we used an ELISA to detect the expression of the four cytokines in the uterine flush samples. Results showed higher IP-10 and VEGF levels in uterine flush samples from women with a history of IA(s) than in samples from the control group (*p*=0.037 and *p*=0.032, respectively; Figure 1).

### 3.3. Differences in the Regulation of HUVEC Migration and Angiogenesis by dNK Cells from Different Groups

**Figure 2:**
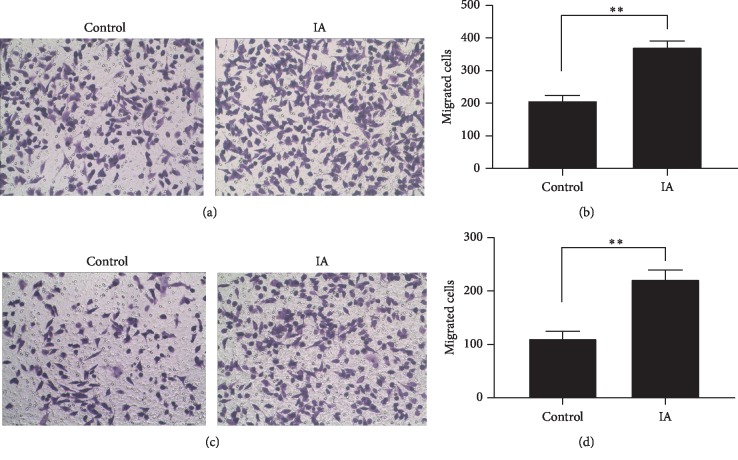
Formation by HUVECs of tube structures of two groups. (a, b) Transwell migration showed the impact of dNK cells on HUVEC (c, d).

Tube formation and Transwell migration assays were performed to evaluate the influence of dNK cells on angiogenesis. The dNK cells isolated from IA and control groups were cocultured with HTR-8/SVneo to imitate the uterine environment. Next, the supernatants were cocultured with HUVEC to facilitate tube formation. The dNK cells from women with a history of early pregnancy were observed to enhance the angiogenesis of HUVECs. Tube formation was significantly enhanced in the IA group compared to the control (*p* < 0.01; Figures 2(a) and 2(b)). The migration activity of HUVECs from the IA group was significantly enhanced compared to that in the control group (*p* < 0.01; Figures 2(c) and 2(d)).

### 3.4. Difference in the Regulation of Invasion and Migration of HTR-8/SVneo Cells by dNK Cells from Different Groups

**Figure 3:**
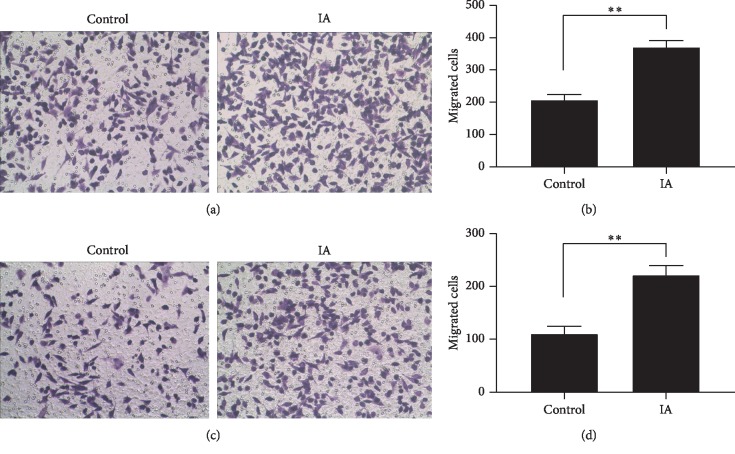
Transwell migration (a, b) and invasion (c, d) of HTR-8/SVneo of two groups.

Transwell migration and invasion assays were performed to evaluate the influence of dNK cells on the invasion and migration abilities of HTR-8/SVneo cells. The dNK cells obtained from IA and control groups were harvested and cocultured with HTR-8/SVneo cells. The dNK cells from women with a history of IAs showed enhanced invasiveness and migration of HTR-8/SVneo cells. The migration activity of HTR-8/SVneo cells from the IA group was better than that in the control group (*p* < 0.01; Figures 3(a) and 3(b)). Transwell invasion assays showed a significant increase in invasiveness in the IA group compared to the control group (*p* < 0.01; Figures 3(c) and 3(d)).

## 4. Discussion

The maternal-fetal interface consists of decidual immune cells, decidual stromal cells, and trophoblast cells. It has been reported that the crosstalk among these cells may contribute to the success of pregnancy [17]. Dysregulation of maternal-fetal interface can lead to many pregnancy disorders. Moreover, several studies have demonstrated the role of cytokines in the maintenance of pregnancy.

dNK cells participate in spiral artery remodeling with trophoblasts [18]. It has been demonstrated that dNK cells regulate trophoblast invasion by producing IP-10. In addition, dNK cells secrete PLGF and VEGF to induce vascular growth [19, 20]. IFN-*γ* may have a positive effect on endothelial integrity. It was confirmed that in early pregnancy, dNK cells are in close contact with the trophoblast and promote invasion and angiogenesis. Many cytokines are secreted due to this cellular interaction [21]. The coculture of dNK and trophoblast cells leads to the production of cytokines such as VEGF and IP-10 [22]. IP-10 has a positive impact on trophoblast invasion, but the mechanism underlying this phenomenon has not yet been elucidated. VEGF is an angiogenesis factor, which is secreted by both decidual and dNK cells [19, 23].

The primary finding of present study is that dNK cells may secrete cytokines (IP-10, VEGF) related to angiogenesis and trophoblast invasion, which contribute to successful pregnancy. The experiments on cell morphology also verified the promotive effect of dNK cells on pregnancy. Besides, we would like to explore as to when the training of dNK cells begins. Our findings provide a further understanding of the role of dNK cells in pregnancy. The history of an early pregnancy might train uterine NK cells to exert a protective effect in a subsequent pregnancy. However, due to the limited sample size, further studies are needed to support these conclusions.

Complete gestation and delivery processes were reported to have a positive influence on the success of subsequent pregnancy. Therefore, in order to eliminate the bias from the history of gestation, women with a history of delivery were excluded. This criterion allowed us to better evaluate the influence of early pregnancy by analyzing decidual tissues that were obtained from IA in first trimester participants. Besides, considering the potential side effects of IA, in the observation group, we only enrolled women with a history of one or two IAs.

One may question that in vitro culture can possibly alter cytokine expression of dNK cells. Hence we collected and analyzed uterine flushing samples by ELISA. The results showed that the four target cytokines (IFN-*γ*, IP-10, VEGF, and PLGF) exhibited the same expression trends between flush samples and the supernatants of dNK cells. Many other types of cells exist at the maternal-fetal interface, the influence of which should not be ignored. However, all things considered, results from the ELISA of supernatants and flush samples confirmed that the in vivo and in vitro environments are comparable.

The present study observed that the level of mRNA and the protein level of some cytokines were not correlated. We hypothesized that there might be some noncoding-RNAs that regulate the expression of these cytokines. Further studies need to be conducted to explain this phenomenon.

The limitation of our study is that we only performed in vitro analysis. We used the HTR-8/SVneo cell line to verify the contribution of dNK cells to placental progress. The HTR-8/SVneo cell line used in our research was derived from the first trimester of gestation, which is somewhat different from actual in the human maternal-fetal interface.

Considering the side effects of IA, we chose the decidual tissue from patients with a history of only one or two IAs to avoid inflammation or endometrial damage. Moreover, we only explored the function of dNK cells. The potential role of decidual stromal cells and the possible interaction between cells in the maternal-fetal interface were not explored in this study. Our results demonstrated that the dNK cell may affect the HUVEC angiogenesis. However, the mechanism remains unknown. Further investigations should be conducted to validate the relationship between the number of early pregnancies and the effect on subsequent pregnancies.

## 5. Conclusion

This study suggests that a history of early pregnancy has a training effect on dNK cells. These trained dNK cells can promote angiogenesis and trophoblast invasion and migration by promoting the production of certain cytokines.

## Figures and Tables

**Table 1:** The primer sequences for Real-time PCR.

Primer set	Forward (5′ to 3′)	Reverse (5′ to 3′)
IP-10	ACCTGCATCAGCATTAGTAATCAAC	GATGGCCTTCGATTCTGGAT
IFN-*γ*	AAAGAGTGTGGAGACCATCAAGGAA	GGATGAGTTCATGTATTGCTTTGCG
PLGF	CTCGTCAGAGGTGGAAGTGGTA	CGCTGGGGTACTCGGACA
VEGF	CAGATTATGCGGATCAAACCTCACC	CACAGGGAACGCTCCAGGACTTAT
18srRNA	GTAACCCGTTGAACCCCATT	CCATCCAATCGGTAGTAGCG

## Data Availability

The data used to support the findings of this study are available from the corresponding author upon request.
