# Erratum to “Human Uterine Decidual NK Cells in Women with a History of Early Pregnancy Enhance Angiogenesis and Trophoblast Invasion”

**DOI:** 10.1155/2020/5024326

**Published:** 2020-06-13

**Authors:** Ningyi Jia, Jian Li

**Affiliations:** Beijing Obstetrics and Gynecology Hospital, Capital Medical University, Beijing, China

In the article titled “Human Uterine Decidual NK Cells in Women with a History of Early Pregnancy Enhance Angiogenesis and Trophoblast Invasion” [1], the published version of Figure 2 does not match the version in the manuscript. The corrected figure is shown below and is listed as Figure 1.

## Figures and Tables

**Figure 1:**
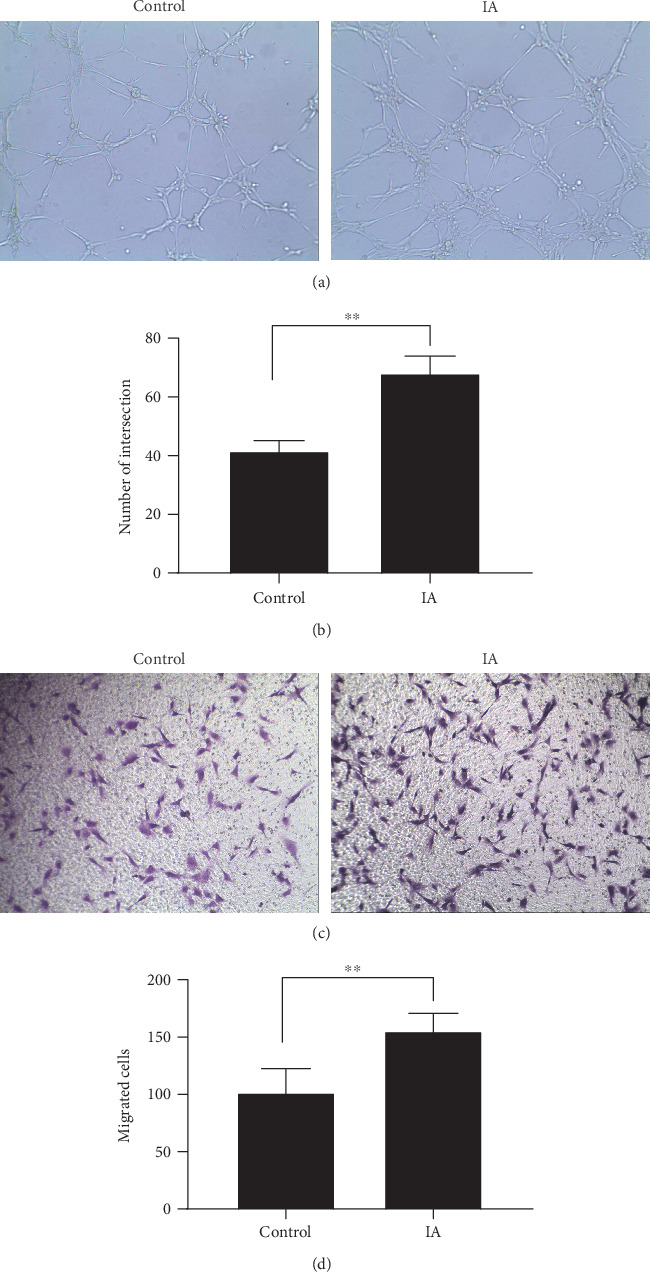
Formation by HUVECs of tube structures of two groups. (a, b) Transwell migration showed the impact of dNK cells on HUVEC (c, d).
